# Balancing Damage via Non-Photochemical Quenching, Phenolic Compounds and Photorespiration in *Ulva prolifera* Induced by Low-Dose and Short-Term UV-B Radiation

**DOI:** 10.3390/ijms23052693

**Published:** 2022-02-28

**Authors:** Yi Zhong, Jinhui Xu, Xinyu Zhao, Tongfei Qu, Chen Guan, Chengzong Hou, Xuexi Tang, Ying Wang

**Affiliations:** 1 College of Marine Life Sciences, Ocean University of China, Qingdao 266003, China; zhongyihaha@foxmail.com (Y.Z.); 17854337097@163.com (J.X.); xyzhao331@gmail.com (X.Z.); tongfeiqu@163.com (T.Q.); guanchen@stu.ouc.edu.cn (C.G.); hcz@stu.ouc.edu.cn (C.H.); 2 Laboratory for Marine Ecology and Environmental Science, Qingdao National Laboratory for Marine Science and Technology, Qingdao 266273, China

**Keywords:** non-photochemical quenching, phenolic compounds, photorespiration, balancing damage, ultraviolet-b radiation, *Ulva prolifera*

## Abstract

The Yellow Sea green tide (YSGT) is the world’s largest transregional macroalgal blooms, and the causative species *Ulva prolifera* (*U. prolifera*) suffers from ultraviolet-b radiation (UVBR) during the floating migration process. Previous study confirmed that *U. prolifera* displayed a wide variety of physiological responses characterized as acclimation to UVBR, while the response mechanisms against low-dose and short-term radiation (LDSTR) are not clear. A study with photosynthetically active radiation (PAR) and UVBR was designed: normal light (NL: 72 μmol photons m^−2^ s^−1^), NL+0.3 (UVBR: 0.3 W·m^−2^), and NL+1.6 (UVBR: 1.6 W·m^−2^). The results showed that high-dose UVBR inhibited photosynthesis in thalli, especially under long-term exposure, while a variety of physiological responses were observed under LDSTR. The inhibition of photosynthesis appeared to be ameliorated by the algae under LDSTR. Further analysis showed that *U. prolifera* achieved balancing damage by means of non-photochemical quenching (NPQ), accumulation of phenolic compounds coupled with the ASA-GSH cycle involved in the antioxidant process and enhanced photorespiratory metabolism under LDSTR. This study provides new insights into the balancing damage mechanisms of *U. prolifera* under LDSTR, enabling the thalli to adapt to the light conditions during the long duration and distance involved in floating migration.

## 1. Introduction

Intertidal macroalgae grow in the intertidal zone and usually undergo both submerged and exposed dry-out environmental changes with the tides. In the vertical distribution, solar radiation is more variable [1]. Shallow-growing or dried-out macroalgae are exposed to strong solar radiation that exceeds the energy requirements provided by their photosynthesis for assimilation [2]. It is well known that solar radiation reaching the Earth’s surface contains ultraviolet-b (UV-B). The thinning of the ozone layer has led to more UV-B radiation (UVBR) reaching the ground. Although UVBR accounts for only a small proportion of the total solar radiation, it has significant ecological and biological effects [3,4]. UVBR causes a decrease of photosynthetic performance that varies according to species, depth of growth, and UV-B penetration at the site of collection [5]. Studies on the harmful effects of high doses of UVBR on algae have been described in numerous research reports, including its effects on growth and development, productivity, antioxidant systems, and DNA damage [6,7].

The resistance strategy of macroalgae under UVBR involves the induction of photoprotective mechanisms and the synthesis of screening pigments involving secondary metabolites [8]. Photoprotective mechanisms ensure that excessive energy absorbed by photosystem II (PSII) is dissipated as thermal radiation, thereby avoiding photodamage [8]. Under short-term UVBR, macroalgae improve heat dissipation by changing the energy transport efficiency from the light capture complex to the reaction center, for example, the non-photochemical quenching (NPQ) induced by the accumulation of the light-harvesting complex (LHC) superfamily members LHC stress-related (LHCSR) and photosystem II subunit S (PSBS) [9]. Studies have shown that certain macroalgae exhibit resistance under UVBR [10]. The functioning of this resistance process can be protected against inhibition by reactive oxygen species (ROS) via antioxidative substances and enzymes in the chloroplast [11]. In addition, the concentrations of phenolic compounds in thalli were found to increase under UVBR [12]. In addition to acting as pigments in thalli, phenolic compounds have a vast array of other biological functions, especially in stress resistance [13].

Many studies have shown that the amelioration of photoinhibition will be delayed after exposure to excess UVBR [14]. The main effect of UVBR on algae is photoinhibition [15]. However, it is noteworthy that specific resistance exists in some macroalgae at moderate levels of UVBR [16]. For macroalgae, it is well known that the species-specific resistance to experimental UVBR exposure can be related to the depth distribution at the community level. Some studies have demonstrated that in the macroalgae *Dictyota dichomata*, recovery of photoinhibition was significantly delayed in samples collected in high UV environments if the natural UV-B wavelength range was removed from the solar spectrum [16]. This was later confirmed under simulated sunlight conditions in different aquatic plants in New Zealand [17]. Some scholars have reported a positive effect of UVBR on phytoplankton growth and abundance: certain taxa were more abundant after the UVBR treatment [18]. These studies indicated an important and unknown regulatory impact of UVBR on the light defensive mechanisms in these species, but the mechanisms are as yet unclear. Effects of low doses of UVBR have been recognized in higher plants, especially in the synthesis of anthocyanins or flavonoids induced by UVBR [19]. Also, UVBR may promote the synthesis of chlorophylls and carotenoids in higher plants as well as the expansion of the hypocotyl of germinating seeds [20]. Therefore, we hypothesized that an ameliorating effect of UVBR may exist in some macroalgae, especially under low-dose excitation.

As the causative species of the Yellow Sea green tide, the floating *U. prolifera* are exposed to more hazardous diel solar radiation on the sea surface during their floating migration process [21,22]. Due to its inflatable structure [22], the thalli often float on the sea surface and are thus exposed to drastic changes in PAR and UVBR [23]. As observed in our previous study, the recovery of physiological state was observed under low solar radiation during a diel cycle [21,22]. We infer the ability of balancing damage in the thalli of *U. prolifera* based on the previous studies [24]. In this study, we discuss the ability of balancing damage in *U. prolifera* induced by low-dose UVBR from the perspective of physiological indexes and metabolomics.

## 2. Results

### 2.1. Changes of F_v_/F_m_, Y(II), Y(NPQ), and Y(NO) in U. prolifera under NL+0.3 and NL+1.6

**Figure 1:**
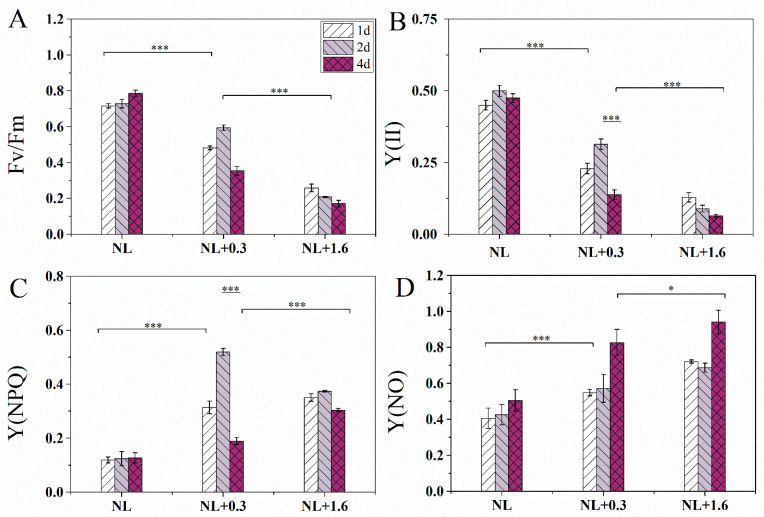
Variation of F_v_/F_m_ (**A**), the effective quantum yield of PSII [Y(II)] (**B**), and the quantum yield of the regulated energy dissipation in PSII [Y(NPQ)] (**C**), the quantum yield of non-regulated energy dissipation in PSII [Y(NO)] (**D**) in *U. prolifera* under NL+0.3 or NL+1.6. Statistical significance is indicated by * (*p* < 0.05) and *** (*p* < 0.001).

The F_v_/F_m_ values under normal light (NL) were significantly higher than those under NL+0.3 and NL+1.6(one-way ANOVA, *p* < 0.001, Figure 1A). Under NL+1.6, F_v_/F_m_ was lower than those of NL and NL+0.3 (one-way ANOVA, *p* < 0.001). F_v_/F_m_ under NL+0.3 at 2 d was significantly high than that at 1 d (one-way ANOVA, *p* < 0.001). For Y(II), similar trends as for F_v_/F_m_ were observed in Figure 1B. The changes in Y(NPQ) were shown in Figure 1C. A clearly increasing tendency under NL+0.3 at 2 d was observed (one-way ANOVA, *p* < 0.001), and then, a clear decline was detected under NL+0.3 at 4 d (one-way ANOVA, *p* < 0.001). The value did decrease under NL+1.6 compared with the other treatments. Y(NO) was higher at 4 d than in other groups (one-way ANOVA, *p* < 0.05, Figure 1D).

### 2.2. Transmission Electron Microscopy Showed Changes in U. prolifera under NL+0.3 and NL+1.6

**Figure 2:**
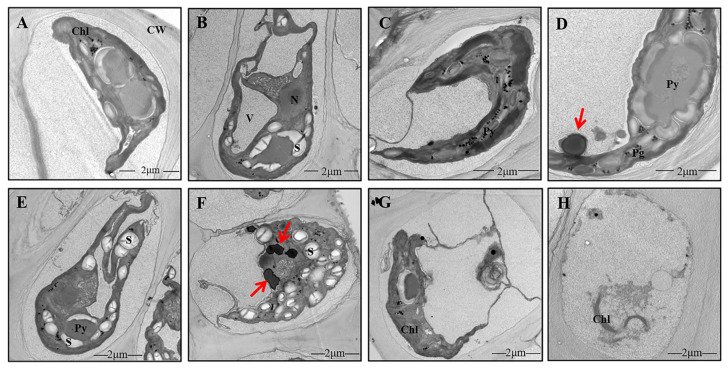
Details of the ultrastructure of the cells of *U. prolifera* under NL+0.3 or NL+1.6. (**A**): Long and narrow chloroplast lobes under NL. (**B**): Cells of *U. prolifera* under NL+0.3 at 1 d. (**C**): Cells of *U. prolifera* under NL+1.6 at 1 d. (**D**–**E**): Cells of *U. prolifera* under NL+0.3 at 2 d. (**F**): Cells of *U. prolifera* under NL+1.6 at 2 d. (**G**): Cells of *U. prolifera* under NL+0.3 at 4 d. (**H**): Cells of *U. prolifera* under NL+1.6 at 4 d. Chl chloroplast; N nucleus; Py pyrenoid; S starch; CW cell wall; V vacuole; electron-dense bodies (arrows).

Under NL, the cells of *U. prolifera* showed a high degree of vacuolation and narrow chloroplast lobes (Figure 2A). There were two pyrenoids that were surrounded by starch. There were no obvious electron-dense bodies. The nucleus under NL+0.3 was intact and clearly visible, and the intact pyrenoid structure was still visible in the chloroplast and was surrounded by starch (Figure 2B). No electron-dense bodies were found (Figure 2B). Under NL+1.6, the structure of pyrenoids was incomplete at 1 d due to the lack of a starch sheath (Figure 2C). The pyrenoids were clearly visible under NL+0.3 at 2 d (Figure 2D). Compared with the changes at 1 d, high amounts of starch accumulated around the pyrenoids, and there were clearly visible electron-dense bodies at 2 d under NL+0.3 (Figure 2E). Under NL+1.6, chloroplast thylakoids were dispersed, and stroma starch grains were accumulated. The morphology of pyrenoids changed, having an incomplete starch sheath. More electron-dense bodies appeared under NL+1.6, and these were sometimes massive (Figure 2F). The results of the morphological observation and electron microscopy showed that the structure of thylakoids became looser at 4 d. The amount of stroma starch decreased significantly, and electron-dense bodies disappeared under NL+1.6 (Figure 2H). Under NL+0.3, stroma starch grains and electron-dense bodies were decreased significantly at 4 d (Figure 2G).

### 2.3. Changes of the Antioxidant System in U. prolifera under NL+0.3 and NL+1.6

**Figure 3:**
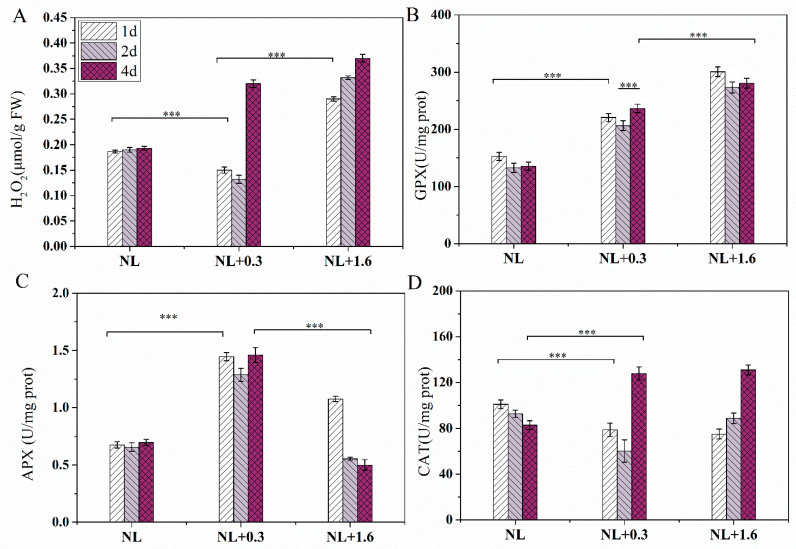
Changes in the antioxidant system of *U. prolifera* under NL+0.3 or NL+1.6: (**A**) H_2_O_2_ content (**B**) GPX activity, (**C**) APX activity, (**D**) CAT activity. Statistical significance is indicated by *** (*p* < 0.001).

A significant difference in H_2_O_2_ content was observed under NL+0.3 and NL+1.6 conditions. H_2_O_2_ content was reduced significantly at 1 d, reduced continuously at 2 d, and significantly increased at 4 d under NL+0.3 compared with the NL (one-way ANOVA, *p* < 0.05). Unlike NL+0.3, a significant increasing trend was observed under NL+1.6 (one-way ANOVA, *p* < 0.001, Figure 3A).

The effects of NL+0.3 and NL+1.6 on the antioxidant enzymes were shown in Figure 3. The activity of GPX was increased significantly at 1 d, decreased slightly at 2 d, and then increased at 4 d under NL+0.3 (one-way ANOVA, *p* < 0.001, Figure 3B). Similarly, the activity of GPX was increased significantly at 1 d, decreased slightly on 2 d, and then did not change significantly at 4 d under NL+1.6 (one-way ANOVA, *p* < 0.001). The level of enzyme activity under NL+1.6 was higher than that under NL+0.3. Similarly, the APX activities under different treatments significantly increased under NL+0.3 (one-way ANOVA, *p* < 0.001, Figure 3C); these were 3.03-, 2.82-, and 2.94-fold the NL values at 1 d, 2 d, and 4 d, respectively. The APX activity under NL+1.6 was at a low level at 2 d and then decreased at 4 d (one-way ANOVA, *p* < 0.001). The CAT located in peroxisomes can catalyze the decomposition of H_2_O_2_. Under NL+0.3 and NL+1.6, the activity of CAT was decreased significantly at 1 d and 2 d and was increased significantly at 4 d (one-way ANOVA, *p* < 0.001, Figure 3D).

### 2.4. Metabonomics in U. prolifera under NL+0.3 and NL+1.6

**Figure 4:**
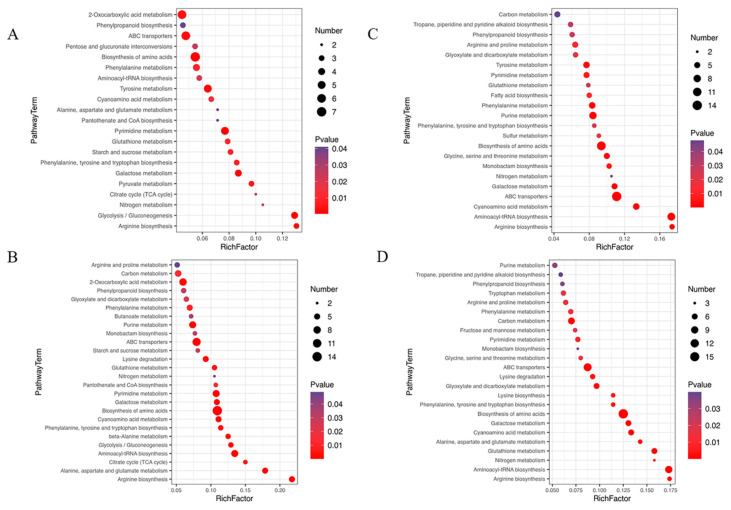
Bubble diagram of differential metabolic pathways in *U. prolifera* cells under NL+0.3 and NL+1.6. (**A**–**D**): 0.3–1~NL-1 (under NL+0.3 at 1 d–under NL at 1 d), 1.6–1~NL-1 (under NL+1.6 at 1 d–under NL at 1 d), 0.3–2~NL-2 (under NL+0.3 at 2 d–under NL at 2 d), 1.6–2~NL-2 (under NL+1.6 at 2 d–under NL at 2 d). The shade of the dot color indicates the size of the *p* value, which represents the significance of the degree of influence on a certain pathway. The red color indicates that the smaller the *p* value, the more significant the effect on the pathway. The purple color indicates a higher *p* value. A larger area of the point indicates that more differential metabolites are involved in the metabolic pathway. The figure shows the first 20 metabolic pathways in the enrichment metabolic pathway with a *p* value < 0.05.

Compared with the groups under NL, 55 differential metabolic pathways were identified under NL+0.3 at 1 d (Figure 4A), including glycolysis, amino acid biosynthesis, and galactose metabolism. Meanwhile, 61 different metabolic pathways were identified, including amino acid biosynthesis, phenylalanine, tyrosine and tryptophan biosynthesis, glyoxylate and dicarboxylate metabolism, and phenylpropanoid biosynthesis, under NL+1.6 at 1 d (Figure 4B).

Compared with the groups under NL, 53 different metabolic pathways were identified, including glyoxylate and dicarboxylate metabolism, phenylalanine, tyrosine and tryptophan biosynthesis, phenylpropanoid biosynthesis, and glutathione metabolism, under NL+0.3 at 2 d (Figure 4C). Compared with the groups under NL, 63 different metabolic pathways were identified, including carbon metabolism, alanine, aspartate, and glutamate metabolism, and phenylalanine, tyrosine, and tryptophan biosynthesis, under NL+1.6 at 2 d (Figure 4D).

**Figure 5:**
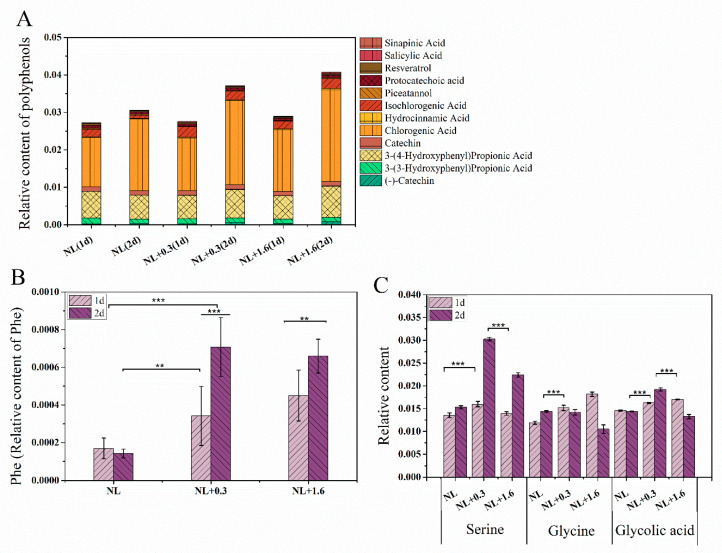
The variation in relative contents of phenolic compounds (**A**), phenylalanine (**B**), and relative content of main photorespiration metabolites (**C**) in *U. prolifera* under NL+0.3 or NL+1.6. Statistical significance is indicated by ** (*p* < 0.01), and *** (*p* < 0.001).

In this study, 12 polyphenolic compounds were identified, three catechins and their derivatives, seven phenylpropanoids, and two stilbenes. Chlorogenic acid belongs to phenylpropanoids and has a high relative content, followed by 3-4-dihydroxypropionic acid. Metabonomics analysis showed that the phenylpropanoid metabolic pathway was enhanced under NL+0.3 at 2 d, which promoted the accumulation of phenylpropanoid substances in *U. prolifera*. Compared with that at 1 d, the relative content of chlorogenic acid was up-regulated at 2 d. Compared with other samples, the relative content of chlorogenic acid was up-regulated to the highest level at 2 d under NL+1.6. Similarly, the relative content of 3-4-dihydroxypropionic acid was up-regulated under NL+0.3 and NL+1.6 at 2 d (Figure 5A). Detailed information regarding all differential metabolic pathways was summarized in Supplementary Table S1 (see Supplementary Materials).

The relative content of phenylalanine (Phe) was also significantly up-regulated under NL+0.3 and NL+1.6 at 2 d (Figure 5B).

### 2.5. Changes of DHAR Activity, GSH and DHA Levels in U. prolifera under NL+0.3 and NL+1.6

**Figure 6:**
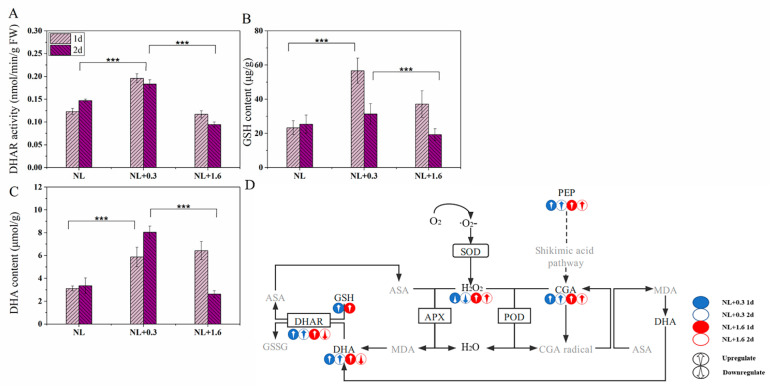
Variation of DHAR activity (**A**), GSH (**B**), and DHA (**C**) contents. Hypothetical phenolic compounds coupled with the ASA−GSH cycle involved in the antioxidant process (**D**) in *U. prolifera* under NL+0.3 or NL+1.6. Statistical significance is indicated by *** (*p* < 0.001).

The activity of DHAR in *U. prolifera* was affected by the different treatments. Compared with that under NL, DHAR activity increased significantly under NL+0.3 (one-way ANOVA, *p* < 0.001, (Figure 6A)). The DHAR activity under NL+1.6 was significantly lower than that under NL+0.3 (one-way ANOVA, *p* < 0.001). Figure 6 also showed the changes in the contents of GSH and DHA in *U. prolifera*. Compared with that under NL, the GSH content under NL+0.3 was increased significantly at 1 d (one-way ANOVA, *p* < 0.001, Figure 6B). This indicated that the GSH content of *U. prolifera* increased sharply under UVBR (Figure 6B). In addition, under NL+0.3 and under NL+1.6 at 1 d, there was a higher content of DHA (Figure 6C).

### 2.6. Changes of the Activities of Rubisco, GO, SGAT, and GLYK and the Contents of HPA and 3-PG in the Photorespiration in U. prolifera under NL+0.3 and NL+1.6

**Figure 7:**
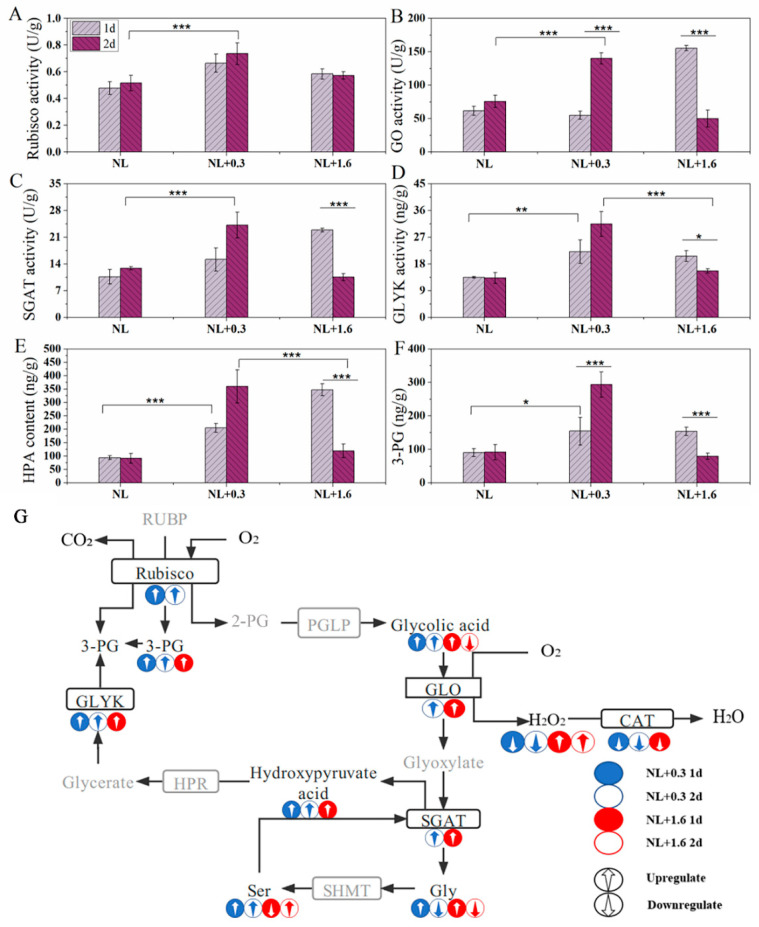
The activities of Rubisco (**A**), GO (**B**), SGAT (**C**), and GLYK (**D**) and content of HPA (**E**) and 3-PG (**F**). Photorespiratory metabolic map (**G**) of *U. prolifera* under NL+0.3 or NL+1.6. Statistical significance is indicated by * (*p* < 0.05), ** (*p* < 0.01), and *** (*p* < 0.001).

Under NL+0.3, the activity of Rubisco increased significantly at 2 d compared with that at 1 d (one-way ANOVA, *p* < 0.001, Figure 7A). GO, SGAT, and GLYK are key enzymes in photorespiration. The activity of GO decreased under NL+0.3 at 1 d (one-way ANOVA, *p* < 0.001) and then increased under NL+0.3 at 2 d (one-way ANOVA, *p* < 0.001) (Figure 7B). SGAT can deaminate serine to form hydroxy pyruvic acid [25]. At 1 d, the activity of SGAT under NL+1.6 was significantly increased (one-way ANOVA, *p* < 0.001), being 2.2 times that under NL, but the change was not significant under NL+0.3. Compared with NL, the activity of SGAT under NL+0.3 was significantly increased at 2 d, but there was no significant change under NL+1.6 at 2 d (Figure 7C). Glycerate kinase catalyzes the formation of 3-phosphoglycerate (3-PG) from glyceric acid, and 3-PG then enters the Calvin cycle [26]. The results showed that the activity of GLYK increased significantly under NL+0.3 at 1 d and then increased continuously at 2 d (one-way ANOVA, *p* < 0.001). Under NL+1.6, the activity of GLYK increased at 1 d compared with NL, being 0.7 times higher at 2 d than at 1 d(Figure 7D). Variation in the content of hydroxypyruvic acid (HPA) was illustrated in Figure 7E. The results showed that the content increased significantly to 2.23 and 3.79 times the NL content under NL+0.3 at 1 d and 2 d, respectively, indicating that production of HPA was promoted under NL+1.6. The content of HPA was significantly increased under NL+1.6 at 1 d, but at 2 d, there was no significant difference between the treatments (one-way ANOVA, *p* > 0.05). The change trend of 3-PG content was basically consistent with that of HPA(Figure 7F).

Figure 5C shows the relative contents of serine, glycolic acid, and glycine in *U. prolifera* under different treatments. Serine, glycolic acid, and glycine are important intermediates in photorespiration. Metabonomics results showed that at 1 d, the relative content of glycolic acid under NL+1.6 was upregulated, while the relative content of glycolic acid under NL+0.3 at 2 d was higher than that under NL. Under NL+1.6, the relative content of glycine was increased significantly at 1 d and then decreased significantly at 2 d. Interestingly, at 2 d, under NL+0.3, the relative content of serine was significantly upregulated. Combined with the increase of SGAT enzyme activity and HPA content, this suggests that the respiratory metabolism was enhanced at this time.

## 3. Discussion

Our previous study showed that excessive ROS were produced in *U. prolifera* exposed to high doses of UVBR and could cause irreversible damage [27]. Similarly, in the present study, we also observed that UVBR inhibited photosynthesis in *U. prolifera*, especially under high-dose and long-term exposure. Interestingly, *U. prolifera* exhibited a variety of physiological changes characterized by acclimation to solar radiation under low-dose and short-term UVBR. Hence, we further analyzed various changes in photosynthetic and antioxidative physiology, the subcellular structure, and metabolomics in thalli under this stress.

### 3.1. Photosynthesis Was Influenced in U. prolifera under NL+0.3 and NL+1.6

Light intensity variation on the sea surface will affect the physiological activities of *U. prolifera*, and the thalli have a variety of mechanisms to resist high solar radiation, especially for UVBR [28]. Under NL+0.3 radiation, F_v_/F_m_ and Y(II) showed an upward trend at 2 d, indicating that low-dose and short-term UVBR may ameliorate the physiological characteristics of PSII (Figure 1). NPQ occurs in various plants, including vascular plants, mosses, and algae [29,30,31]. NPQ plays an important role during the process of photoprotection under UVBR. In this study, Y(NPQ) increased significantly under NL+0.3. Y(NO) was relatively stable under short-term radiation under NL+0.3 (Figure 2C). This implies that the NPQ mechanism plays an important role in *U. prolifera* under low-dose UVBR. Research has shown that the improvement effect of UVBR on tropical marine *Gracilaria* at 21 °C was due to the higher recovery of the highest quantum yield after moderate UV-B treatment [32]. We speculated that low-dose UVBR can significantly ameliorate the physiological properties of PSII in *U. prolifera* exposed to drastic changes during a diel cycle.

### 3.2. Structural Alterations in U. prolifera under NL+0.3 and NL+1.6

In this study, some ultrastructural changes were observed after radiation, namely, changes in the amounts of stroma starch grains and electron-dense bodies in the chloroplasts. In this study, the amount of stroma starch decreased on 1 d under NL+1.6 (Figure 2C), but stroma starch accumulated heavily, and the starch sheath was decreased at 2 d (Figure 2F). Under NL+0.3, stroma starch increased at 2 d (Figure 2D,E). The increase of photosynthetic activity provides guarantee for the formation of starch (Figure 1A,B). Studies have shown that under UVBR, plants usually experience metabolic changes, resulting in the accumulation of carbohydrates and the redistribution of organic carbon [33,34].

The pyrenoids are usually surrounded by a starch sheath [35]. Under NL+1.6 at 2 d, the starch sheath around the pyrenoids was destroyed. Although little was known about the mechanism of starch sheath formation, there was evidence for the independent regulation of stroma starch and pyrenoid starch synthesis [36]. The starch redistribution between pyrenoid starch and stroma starch observed in *Chlamydomonas reinhardtii* was unrelated to the change in total starch content [36]. These results showed that pyrenoid starch metabolism is different from that in the matrix. Under NL+1.6 treatment, Rubisco activity increased and GO activity decreased, indicating that carboxylation functions were enhanced and that thalli began to adapt to efficient carbon fixation, resulting in an increase in the amount of stroma starch, but there was no significant increase in pyrenoid starch.

The most prominent structures that have been attributed to photoprotection under UVBR are the electron-dense bodies [37,38]. These structures have been found in field samples of an Arctic strain [37]. Pichrtová et al. speculated that these bodies, with a diameter of 400–600 nm, contain phenolics [39]. Here, we showed that they could be found in the experimental group at 1 d, but under NL+1.6, these electron-dense bodies tended to aggregate, as shown in Figure 2F. This observation was consistent with the increase of phenolic compounds in *U. prolifera* detected by high performance liquid chromatography. However, we are still unable to provide evidence concerning the chemical properties of these compartments, although they react strongly with osmium tetroxide, resulting in the appearance of electron density.

### 3.3. Analysis of Antioxidant Enzyme Activity and Phenolic Compounds in U. prolifera under NL+0.3 and NL+1.6

In this study, the H_2_O_2_ content decreased continuously under NL+0.3 at 1 d and 2 d. The content of H_2_O_2_ increased continuously under NL+1.6. It can be seen that the antioxidant system played an important role in removing excess H_2_O_2_ under NL+0.3 (Figure 3) and that the thalli suffered more serious damage under NL+1.6. Research has shown that ROS is an important marker of cellular response to environmental stresses [40,41]. Photosynthesis is an important factor in the production of H_2_O_2_, which is related to the environmental adaptability in *U. prolifera*. H_2_O_2_ plays an important role in the activation of the antioxidant system and secondary signal pathway under UVBR [27]. APX is located in chloroplasts that lack CAT, and it is an important part of the GSH-ASA cycle in algae. The results of enzyme activity analysis showed that APX activity gradually increased under the treatments. These results showed that APX had a significant protective effect on the algae under UVBR. This is consistent with the conclusion that APX has the strongest affinity for H_2_O_2_ and can effectively remove it [42]. In addition, the relevant research showed that APX with high activity can compensate for the decrease in CAT activity, thus preventing thalli from being subjected to oxidative stress [43]. GPX is a sulfhydryl peroxidase that can remove H_2_O_2_, organic hydroperoxides, and lipid peroxides in algae, blocking further damage by ROS to the algae [44]. The results showed that the activity of this enzyme was increased under UVBR stress, reaching a maximum under NL+1.6 at 1 d. The results for enzyme activity showed that the antioxidant enzyme system played an important role in the algae’s resistance to oxidative stress.

Research has shown that plants can scavenge ROS by relying on phenolic secondary metabolites as substrates or directly as ROS scavengers; this is also an important defense mechanism of plants under UVBR [45]. Compared with the content under NL, we observed that the relative content of phenolic compounds was significantly increased under other treatments and that the content was higher at 2 d than at 1 d (Figure 5A). The relative content of chlorogenic acid under NL+0.3 and NL+1.6 at 2 d was much higher than that of other groups.

Under UVBR, not all polyphenols increase at the same rate in plants from lower liverworts to higher gymnosperms [46]. Research has shown that phenolic compounds and antioxidant enzymes may have good complementary ability in antioxidation [47]. The defense mechanisms in *U. prolifera* against UVBR may differ under NL+0.3 and NL+1.6 conditions. With the increase of DHAR activity and APX activity, the contents of GSH and DHA increased significantly at 1 d, indicating that the regulation of the antioxidant enzyme ASA−GSH cycle played a major role in the antioxidant system under NL+0.3. Compared to 1 d, the relative content of chlorogenic acid increased significantly at 2 d; the APX activity decreased significantly, and in combination with the decrease in DHAR activity, the DHA content increased. Therefore, we conclude that the regulation of phenolic compounds played a prominent role in the antioxidant system at 1 d under NL+0.3. This shows that the antioxidant capacity may not be consistent with the activities of antioxidant enzymes [48]. Under NL+1.6, DHAR activity was still decreased and APX activity was decreased compared to that at 1 d. Coupled with the decreases in the relative content of Gly and the contents of DHA and GSH, the ASA supplementation response was adversely affected. Due to substrate limitation, this strategy was not sufficient to protect cells in *U. prolifera* from UVBR damage, even if the relative content of chlorogenic acid were increased.

### 3.4. Effects of Photorespiratory Metabolism in U. prolifera under NL+0.3 and NL+1.6

GO is the key enzyme in photorespiration that can catalyze the oxidation of glycolic acid. In this study, there was no significant change in GO activity at 1 d under NL+0.3. This indicated that photorespiration did not play a major role in regulating the activity of *U. prolifera* in this treatment. At 2 d under NL+0.3, the activity of GO was significantly increased.

The activities of SGAT and GLYK were enhanced, and the contents of key metabolites of photorespiration were increased, indicating that the photorespiration metabolism was enhanced at this treatment (Figure 7). SGAT can catalyze glyoxylic acid to synthesize glycine by transamination with serine as a substrate. Photorespiration is the main pathway of glycine synthesis in plants [49]. Compared with the NL, the relative content of glycine was downregulated, and the content of GSH was reduced compared with that at 1 d, indicating that part of the source of GSH is that GR restores GSSG to GSH. Yang et al. (2013) transferred the SGAT *Arabidopsis* gene into *Lemna minor* [50]. The transgenic *Lemna minor* showed higher photochemical efficiency and lower intracellular ROS levels under salt stress, indicating that the SGAT gene could help plants maintain higher photochemical efficiency and lower ROS levels under stress. In this study, the SGAT activity was increased under NL+0.3, indicating that SGAT played an important role in maintaining high photochemical efficiency in *U. prolifera*. Under NL+1.6 at 1 d, GO activity was increased, and the key photorespiration metabolites were enhanced, indicating that photorespiration was enhanced. Under NL+1.6 at 2 d, with the decreasing in GO activity, photorespiration metabolism was weakened, which was not enough to resist the stress of high-dose UVBR. Previous research suggested that Rubisco catalyzes 1 mol of O_2_ and 1 mol of RuBP to produce 1 mol of 2PG and 3-PG in chloroplasts [51]. In this study, we found that GLYK activity and 3-PG content decreased and that Rubisco activity increased, indicating that *U. prolifera* could enhance the accumulation of starch grains under high doses of UVBR. Starch accumulation is considered to be an ideal method of energy storage, and there is no evidence to show that it is related to UVBR tolerance [52].

### 3.5. Balancing Damage via NPQ, Phenolic Compounds and Photorespiration

**Figure 8:**
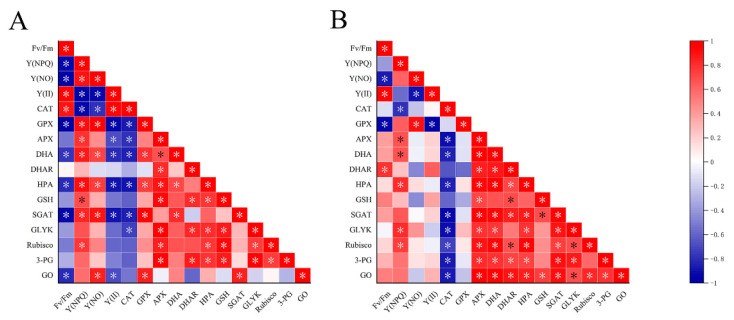
Pearson’s correlation coefficients in the test index in the different groups of *Ulva prolifera*. The color of red represents positive correlation and blue represents negative correlation. (**A**) at 1 d (**B**) at 2 d. Statistical significance is indicated by * (*p* < 0.05).

As a complete organism, various components in the thalli work together under UVBR, but little research has focused on the relationships among NPQ, antioxidant systems and photorespiration. We have studied the correlation among the systems using correlation analysis (Figure 8).

**Figure 9:**
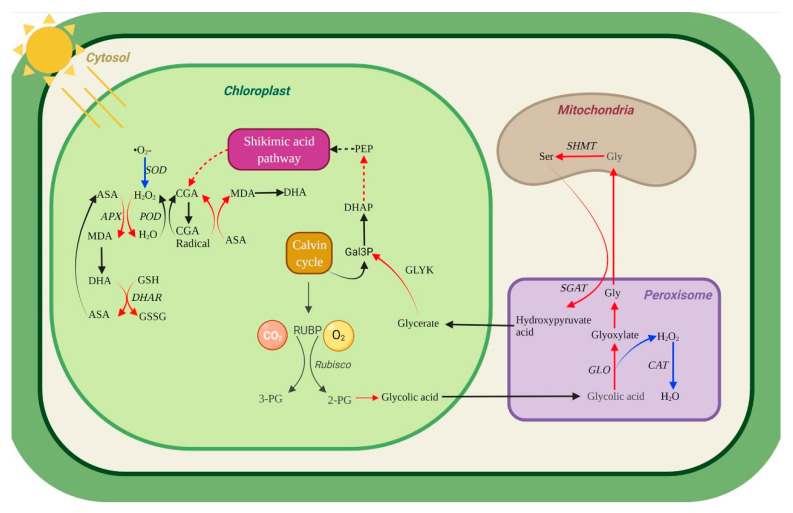
Hypothetical metabolic map in *U. prolifera* under lowdose and shortterm UV-B. Enhanced photorespiration, phenolic compounds metabolism, and the AsA−GSH cycle may contribute to explain the balancing damage mechanism in *U. prolifera* under UVBR stress. The red line represents the enhancement of a metabolic pathway. The blue line indicates that the metabolic pathway is inhibited. Black line, no supporting data on metabolic pathways. Enzymes are represented by italic letters.

In this research, with the increased dose of UV-B radiation, the activities of GO, SGAT and other key photorespiration enzymes in *U. prolifera* were positively correlated with NPQ. NPQ increased and enzyme activity increased, indicating that the photorespiration and NPQ under UVBR stress act on thalli at the same time to dissipate excess light energy. A significantly negative correlation existed between F_v_/F_m_ and GO activity at 1 d. In contrast, the significantly positive correlation existed between them at 2 d. This indicated that photoprotection by photorespiration is efficient at 2 d. This result was consistent with part of 2.6. NPQ ensures the operation of light reaction in the thalli under stresses [53]. Enhanced photorespiration can accelerate the removing of 2-phosphoglycolate, so as to maintain the activity of Calvin cycle [54]. APX was significantly positively correlated with photorespiratory key enzymes, and CAT and photorespiratory key enzymes were negatively correlated. This indicated APX as a key enzyme contained in ASA-GSH plays an important role in scavenging H_2_O_2_ under UVBR in the thalli. At 2 d, there was a significant positive correlation between GSH and GO, indicating that the synthesis of GSH was related to the process of photorespiration. The glycine produced in the process of photorespiration could provide materials for the synthesis of GSH thus increasing the content of GSH in the thalli.Noctor et al. have proved through a series of studies that glycine produced by photorespiration is an important source of glycine in the synthesis of GSH [49]. The positive correlation at 2 d and the negative correlation at 1 d among DHA, DHAR and F_v_/F_m_ were consistent with the changes in DHA content and DHAR activity in this research. This suggested that the *U. prolifera* can maintain photosynthetic activity in the thalli by coordinating multiple enzymes in the ASA−GSH recycle system under UVBR. Meanwhile, *U. prolifera* relied on phenolic compounds as substrates or directly as ROS scavengers, and they played roles in antioxidant together with enzymes in AsA−GSH cycle such as APX. There is a synergistic relationship between them. Therefore, we speculate that NPQ, antioxidant system composed of phenolic compounds and other substances, photorespiration pathway is an important balancing damage mechanism of *U. prolifera* under UVBR (Figure 9).

## 4. Materials and Methods

### 4.1. Species and Treatments

In this study, *U. prolifera* was isolated at Qingdao (China) during a YSGT outbreak in July 2019. To ensure that we were working with *U. prolifera*, we screened the floating algal samples by morphological identification and molecular identification. The seawater for the culture was taken from Jiaozhou Bay, filtered through a 0.45-mm mixed-fiber membrane, and sterilized at 121 °C for 30 min. The thalli were cleaned with a brush using sterile seawater to remove adhering deposits, small herbivores, and epiphytes. The thalli were then transferred to sterile seawater and added to a nutrient solution to form f/2 medium. The culture temperature was set to 20 °C. The light intensity was set to 72 μmol m^−2^ s^−1^ under a 12 h/12 h light/dark period. The radiant intensity of photosynthetically available radiation (PAR) was measured using Apogee MQ-500 handheld quantum meters (Jiangsu Tynoo Corporation, Wuxi, China). In order to suppress the growth of diatoms, germanium dioxide (GeO_2_) at a concentration of 0.5 mg/L was added.

**Figure 10:**
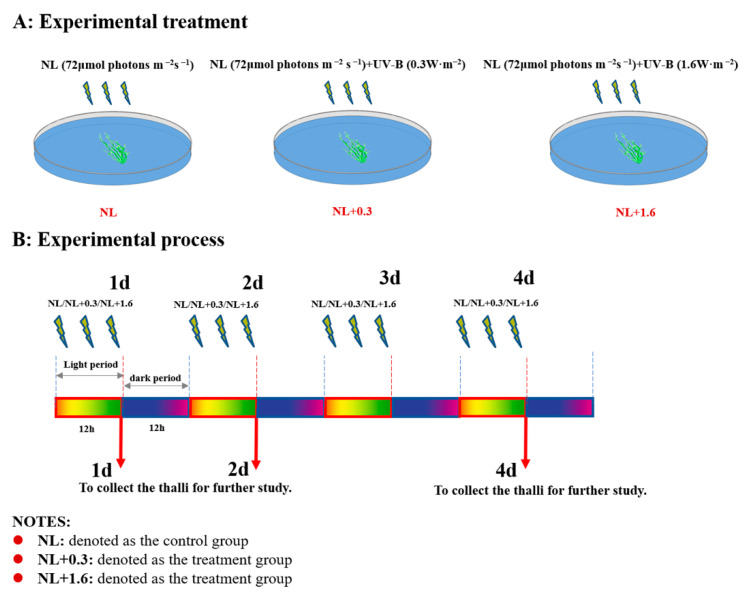
Experimental design of the study. (**A**) Experimental treatment: Setting of experimental grouping and radiation intensity. (**B**) Experimental process: Setting of sampling time and radiation duration.

The YSGT occurs periodically between May and July each year in the Yellow Sea of China [55]. Based on the previous field research, the average UVBR intensity fluctuated between 0.3 W·m^−2^ and 1.6 W·m^−2^ at 8:00–16:00 by using UVBR meters (Juguangyingchuang, Beijing, China). A compound experiment with PAR and UVBR was designed in the laboratory. The UVBR exposure levels were set at 0.3 and 1.6 W·m^−2^, respectively, using UV-B lamps (TL 40W/12RS, Philips, Aachen, Germany) supplemented with 72 μmol m^−2^ s^−1^ PAR. To exclude UV-C radiation, the UV-B lamps were covered with cellulose acetate films (0.12 mm). With the exception of UVBR, PAR which was the same as that used for the culture conditions, was provided to the thalli through cool-fluorescent lamps (TL5-28W, Philips, Shanghai, China). The radiant intensity of UV-B in the lab was measured using the UVBR meters (Photoelectric Instrument Factory of Beijing Normal University, Beijing, China). The thalli were treated by UVBR and PAR from 6:00 to 18:00 each day. The experimental period lasted 4 days. The thalli samples were collected at 18:00 on the 1st day (denoted as 1 d), at 18:00 on the 2nd day (denoted as 2 d), and at 18:00 on the 4th day (denoted as 4 d). The experimental treatments were as follows: normal light (NL: 72 μmol photons m^−2^ s^−1^) (denoted as the control group), normal light+0.3 (NL+0.3, NL: 72 μmol photons m^−2^ s^−1^ and UV-B: 0.3 W·m^−2^) (denoted as the treatment group), and normal light+1.6 (NL+1.6, NL: 72 μmol photons m^−2^ s^−1^ and UV-B: 1.6 W·m^−2^) (denoted as the treatment group) (Figure 10).

### 4.2. Determination of Chlorophyll Fluorescence

Chlorophyll (Chl) fluorescence was determined with a DUAL-PAM 100 (Walz GmbH, Effeltrich, Germany). Cultures were dark adapted for 20 min before determination. The induction curve was constructed under a measuring light from a 620-nm light-emitting diode (LED) and blue actinic light of 100 mmol photons m^−2^ s^−1^ from 460-nm LED arrays for 5 min. Then, saturating light pulses of 300-ms duration and 10,000 mmol photons m^−2^ s^−1^ were delivered to the thalli [53]. The maximum quantum efficiency of PSII (F_v_/F_m_), which is classically used to track changes in photosynthetic performance [56], and it can be obtained from the equation F_v_/F_m_ = (F_m_ − F_0_)/F_m_. The values of F_v_/F_m_ can represent the physiological activity of the sample. In this equation, F_0_ is the minimal fluorescence after dark acclimation, and F_m_ means the maximal fluorescence after saturation flashes in the dark-acclimatized sample. Y(II) is the effective quantum yield of PSII. If, for example, Y(II) = 0.5, this means that 50% of the absorbed quanta have been converted into chemically fixed energy, and the other half of the quanta is dissipated as heat and fluorescence [57]. Y(NPQ) is the quantum yield of the regulated energy dissipation in PSII, and Y(NO) means the quantum yield of non-regulated energy dissipation in PSII [58]. A higher value of Y(NPQ) indicates that the sample retains the physiological means to protect itself by regulation, while a higher Y(NO) value indicates that both photochemical energy conversion and protective regulatory mechanisms are inefficient [58].

### 4.3. Observations via Transmission Electron Microscopy

For transmission electron microscopy, samples were fixed using a standard chemical fixation protocol with modifications [37]. Briefly, cells were fixed in 2.5% glutaraldehyde at room temperature for 1.5 h, rinsed, and post-fixed in 1% OsO_4_ at 4 °C overnight; both fixatives were dissolved in 20 mM cacodylate buffer (pH 7). After dehydration in a series of increasing ethanol concentrations, the cells were embedded in modified Spurr’s resin and heat-polymerized. Ultrathin sections were counterstained with uranyl acetate and Reynold’s lead citrate. Sections were viewed under a transmission electron microscope (TEM, H-7650, Hitachi, Tokyo, Japan) operating at 80 kV. Ten TEM micrographs were examined for each sample.

### 4.4. Measurement of H_2_O_2_ Content

We referred to the method of Ferguson et al. [59] and improved it. The algae were accurately weighed then 0.1 g was added to 0.9 mL of 1 × PBS buffer, and a tissue homogenate prepared by grinding and crushing each group of thalli using a tissue grinder (JXFSTPRP-48L, Shanghaijingxin, Shanghai, China). The next step was centrifuged at 12,000× *g* for 5 min at 4 °C in a high-speed refrigerated centrifuge (GL-20G-II, Anting, Shanghai, China), and the supernatant was obtained. Then, 20% concentration TiCl_4_ in hydrochloric acid solution and concentrated ammonia solution were added to form a titanium peroxide complex. After centrifugation at 14,000× *g* for 15 min, the supernatant was discarded; the precipitate was retained and dissolved in 2 M H_2_SO_4_ solution. The absorbance value was determined at 410 nm. A standard curve was drawn from the absorbance values. All experiments were performed as three replicates; the results were presented as μmol/g FW (fresh weight).

### 4.5. Measurement of CAT, GPX, APX, DHAR, GSH and DHA

Firstly, 0.3 g FW of *U. prolifera* was ground to powder in liquid nitrogen. The algal powder was added to 2.7 mL of 50 mmol/L phosphate buffer (pH = 7.0). The mixture was centrifuged at 12,000× *g* and 4 °C for 10 min. After centrifugation, the supernatant was carefully removed with a pipette. The supernatant was used for the determination of antioxidant enzyme activity of thalli. The precipitate in the tube was discarded.

The CAT, GPX, and APX activities of thalli were determined using a catalase (CAT) activity assay kit, a glutathione peroxidase (GPX) activity assay kit, and an ascorbate-peroxidase (APX) activity assay kit, respectively. The kits were purchased from Nanjing Jiancheng Company (Nanjing, China), and the experimental process was strictly in accordance with the manufacturer’s instructions. The results were presented as U/mg protein.

DHAR activity was measured using DHAR kits (mibio) according to the manufacturer’s instructions. The absorbance (OD) was measured at 265 nm with a microplate reader (Perkin Elmer, Waltham, MA, USA). The results were presented as nmol/min/g FW. The DHA and GSH contents of thalli were determined using a DHA content assay kit and a GSH content assay kit, respectively. The kits were purchased from Beijing Boxbio Science & Technology Co., Ltd. (Beijing, China). The experimental process was performed in strict accordance with the manufacturer’s instructions. The results were presented as μg/g.

### 4.6. Metabolomics Profiling

The sequencing material was derived from *U. prolifera* in the Yellow Sea Green Tide. The radiation system was the same as that in 4.1. After sampling, samples were quickly processed on ice and stored in liquid nitrogen for further GC-MS analysis.

A piece of the tissue (60 mg) was mixed with 360 μL of cold methanol and 40 μL internal standards (0.3 mg/mL 2-chlorophenylalanine inmethanol), and then homogenized using a tissuelyser system (Tissuelyser-192, Shanghai Jingxin Experimental Technology, Shanghai, China). After ultrasonication for 30 min, 200 μL chloroform and 400 μL water were added to the sample. The mixture was vortexed for 2 min and sonicated for 30 min, and then the sample was centrifuged at 14,000 rpm for 10 min at 4 °C. Finally, 400 μL of the supernatant was transferred to a glass sampling vial to vacuum-dry at room temperature. The residue was derivatized using a two-step procedure. First, 80 μL methoxyamine (15 mg/mL in pyridine) was added to the vial, vortexed for 30 s and kept at 37 °C for 90 min followed by 80 μL BSTFA (1% TMCS) and 20 μL n-hexane at 70 °C for 60 min [60].

The samples were analyzed on an Agilent 7890B gas chromatography system coupled to an Agilent 5977A MSD system (Agilent Technologies Inc., Santa Clara, CA, USA). After the data was normalized, redundancy removal and peak merging were conducted to obtain the data matrix. The matrix was carried out principal component analysis (PCA) to observe the overall distribution among the samples and the stability of the whole analysis process. Orthogonal partial least-squares-discriminant analysis (OPLS-DA) and partial least-squares-discriminant analysis (PLS-DA) were utilized to distinguish the metabolites that differ between groups. Variable importance of projection (VIP) values obtained from the OPLS-DA model were used to rank the overall contribution of each variable to group discrimination. A two-tailed Student’s *t*-test was further used to verify whether the metabolites of difference between groups were significant. Differential metabolites were selected with VIP values greater than 1.0 and *p*-values less than 0.05.

All of the differential metabolites were selected by comparing the metabolites using both the multivariate and the univariate statistical methods. The differentially expressed phenolic compounds were screened from the differential metabolites and their types and relative contents were obtained. The relative contents of phenylalanine, serine, glycolic acid and glycine were also screened [61].

### 4.7. Measurement of GO, SGAT, GLYK, HPA and 3-PG

For the pre-treatment procedure for thalli, please refer to Section 4.5. A Boxbio kit (Beijing Boxbio Science & Technology Co., Ltd., Beijing, China) was used to measure the content of GO. The absorbance (OD) of the reaction product was measured at 324 nm by an enzyme labeling instrument. All experiments were performed as three replicates; the results were presented as U/g.

The contents of SGAT, GLYK, HPA, and 3-PG were measured with a microplate reader using an ELISA Kit (Jining Shiye, Shanghai, China) according to the manufacturer’s instructions. The absorbances (OD) of SGAT, GLYK, HPA, and 3-PG were measured at 450 nm with a Perkin Elmer microplate reader, and the contents of SGAT, GLYK (glycerate kinase), HPA, and 3-PG in the samples were calculated according to the standard curve. The units of the SGAT and GLYK activity are U/g and ng/g, respectively. The unit of the HPA and 3-PG content is ng/g.

### 4.8. Statistical Analysis

Data are presented as the means of three replicates (±SD). Results of chlorophyll fluorescence, antioxidant enzyme activity, and other physiological indexes were analyzed by one-way ANOVA (SPSS Inc., Chicago, IL, USA). The differences among data were analyzed with Levene’s test for homogeneity, and all data were homogeneous. Statistical significance is indicated by * (*p* < 0.05), ** (*p* < 0.01), and *** (*p* < 0.001).

## 5. Conclusions

High-dose UVBR inhibited the physiological status of *U. prolifera,* decreasing F_v_/F_m_ and inducing H_2_O_2_, especially under long-term stress. Nevertheless, under low-dose and short-term UVBR, *U. prolifera* showed a variety of effective balancing damage strategies that ensured its adaptation to the environment. From our results the following conclusions can be put forth:

(1) As one of the photoprotective mechanisms, efficient NPQ can ameliorate photosynthetic efficiency of *U. prolifera*.
(2) Significant increases in the amounts of photoprotective structures such as electron-dense bodies were observed in the submicroscopic structure of the algae, observations that were supported by the significantly higher amounts of UV-absorbing phenolic compounds.
(3) *U. prolifera* relies on phenolic compounds as substrates or directly as ROS scavengers, and they play roles in antioxidant together with enzymes in AsA-GSH cycle such as APX.
(4) The enhanced photorespiration pathway can consume excess light energy. Moreover, photorespiration provides 3-PG for the Calvin cycle, thus maintaining high photosynthetic efficiency and ameliorating the ‘balancing damage’ of thalli.

Therefore, in addition to NPQ and the antioxidant system, phenolic compounds as well as photorespiratory pathways play an important role in the balancing damage of *U. prolifera* under low-dose and short-term UVBR exposure. This study provides new insights into the damage-balancing mechanisms of *U. prolifera* under low-dose and short-term UV-B radiation, enabling the thalli to adapt to the light conditions during the long irradiation duration and distance involved in floating migration.

## Data Availability

The data presented in this study are available in the article.

## Supplementary Materials

The following supporting information can be downloaded at: https://www.mdpi.com/article/10.3390/ijms23052693/s1.
