# Supplementary material for: Balancing Damage via Non-Photochemical Quenching, Phenolic Compounds and Photorespiration in Ulva prolifera Induced by Low-Dose and Short-Term UV-B Radiation
Source: Int J Mol Sci. 2022 Feb 28;23(5):2693. doi: 10.3390/ijms23052693 (PMC8911146; doi:10.3390/ijms23052693)
Supplement: Supplementary file 1 [file ijms-23-02693-s001.zip › ijms-1589452-supplementary.pdf]

Supplementary Materials

Table S1. Differential metabolic pathway under different treatments (p value < 0.05).

Treatments / Number	0.3-1~NL-1	1.6-1~NL-1	0.3-2~NL-2	1.6-2~NL-2
1	Glycolysis / Gluconeogenesis	Biosynthesis of amino acids	ABC transporters	Biosynthesis of amino acids
2	Biosynthesis of amino acids	Aminoacyl-tRNA biosynthesis	Aminoacyl-tRNA biosynthesis	Aminoacyl-tRNA biosynthesis
3	Pyrimidine metabolism	Arginine biosynthesis	Biosynthesis of amino acids	ABC transporters
4	Galactose metabolism	ABC transporters	Cyanoamino acid metabolism	Glutathione metabolism
5	Tyrosine metabolism	Alanine, aspartate and glutamate metabolism	Purine metabolism	Cyanoamino acid metabolism
6	Arginine biosynthesis	Pyrimidine metabolism	Arginine biosynthesis	Galactose metabolism
7	ABC transporters	Cyanoamino acid metabolism	Galactose metabolism	Arginine biosynthesis
8	2-Oxocarboxylic acid metabolism	Galactose metabolism	Phenylalanine metabolism	Glyoxylate and dicarboxylate metabolism
9	Pyruvate metabolism	Purine metabolism	Glycine, serine and threonine metabolism	Carbon metabolism
10	Phenylalanine, tyrosine and tryptophan biosynthesis	Glycolysis / Gluconeogenesis	Tyrosine metabolism	Alanine, aspartate and glutamate metabolism
11	Starch and sucrose metabolism	beta-Alanine metabolism	Monobactam biosynthesis	Lysine degradation
12	Phenylalanine metabolism	2-Oxocarboxylic acid metabolism	Pyrimidine metabolism	Lysine biosynthesis
13	Glutathione metabolism	Lysine degradation	Fatty acid biosynthesis	Phenylalanine, tyrosine and tryptophan biosynthesis
14	Cyanoamino acid metabolism	Phenylalanine, tyrosine and tryptophan biosynthesis	Arginine and proline metabolism	Nitrogen metabolism
15	Nitrogen metabolism	Glutathione metabolism	Sulfur metabolism	Pyrimidine metabolism

16	Aminoacyl-tRNA biosynthesis	Citrate cycle (TCA cycle)	Glyoxylate and dicarboxylate metabolism	Phenylalanine metabolism
17	Citrate cycle (TCA cycle)	Phenylalanine metabolism	Phenylalanine, tyrosine and tryptophan biosynthesis	Glycine, serine and threonine metabolism
18	Pentose and glucuronate interconversions	Pantothenate and CoA biosynthesis	Phenylpropanoid biosynthesis	Arginine and proline metabolism
19	Phenylpropanoid biosynthesis	Carbon metabolism	Glutathione metabolism	Tryptophan metabolism
20	Alanine, aspartate and glutamate metabolism	Glyoxylate and dicarboxylate metabolism	Tropane, piperidine and pyridine alkaloid biosynthesis	Fructose and mannose metabolism
21	Pantothenate and CoA biosynthesis	Starch and sucrose metabolism	Nitrogen metabolism	Purine metabolism
22		Phenylpropanoid biosynthesis	Carbon metabolism	Phenylpropanoid biosynthesis
23		Monobactam biosynthesis		Monobactam biosynthesis
24		Butanoate metabolism		Tropane, piperidine and pyridine alkaloid biosynthesis
25		Nitrogen metabolism		
26		Arginine and proline metabolism		
